# Age-dependent changes in clock neuron structural plasticity and excitability are associated with a decrease in circadian output behavior and sleep

**DOI:** 10.1016/j.neurobiolaging.2019.01.025

**Published:** 2019-05

**Authors:** Jack A. Curran, Edgar Buhl, Krasimira Tsaneva-Atanasova, James J.L. Hodge

**Affiliations:** a School of Physiology, Pharmacology and Neuroscience, University of Bristol, Bristol, UK; b Department of Mathematics and Living Systems Institute, University of Exeter, Exeter, UK; c EPSRC Centre for Predictive Modelling in Healthcare, University of Exeter, Exeter, UK

**Keywords:** *Drosophila*, Aging, Circadian rhythms, Sleep, Synaptic plasticity, Electrophysiology, Intrinsic excitability

## Abstract

Aging has significant effects on circadian behavior across a wide variety of species, but the underlying mechanisms are poorly understood. Previous work has demonstrated the age-dependent decline in behavioral output in the model organism *Drosophila*. We demonstrate that this age-dependent decline in circadian output is combined with changes in daily activity of *Drosophila*. Aging also has a large impact on sleep behavior, significantly increasing sleep duration while reducing latency. We used electrophysiology to record from large ventral lateral neurons of the *Drosophila* circadian clock, finding a significant decrease in input resistance with age but no significant changes in spontaneous electrical activity or membrane potential. We propose this change contributes to observed behavioral and sleep changes in light-dark conditions. We also demonstrate a reduction in the daily plasticity of the architecture of the small ventral lateral neurons, likely underlying the reduction in circadian rhythmicity during aging. These results provide further insights into the effect of aging on circadian biology, demonstrating age-related changes in electrical activity in conjunction with the decline in behavioral outputs.

## 1 Introduction

Circadian rhythms describe the near 24-hour cycle in behavior and physiology, driven by the circadian clock, which allow organisms to anticipate daily changes in their environment. Circadian clocks in animals are fundamental biological components responsible for the control of large aspects of physiology and behavior, ranging from the sleep-wake cycle to rhythms in blood pressure (Roenneberg and Merrow, 2016). Remarkably, the fundamental molecular basis of the intracellular clock is well conserved from *Drosophila* to mice and humans (Allada and Chung, 2010). The health consequences of circadian misalignment as a result of our modern lifestyles are dramatic, with links to cancer, depression, and sleep disorders (Menet and Rosbash, 2011, Samuelsson et al., 2018, West and Bechtold, 2015). With increasing human life spans and an aging population, understanding how circadian rhythms change during the aging process is of growing interest and medical relevance, with the population aged over 60 years set to more than double by 2050 (UN, 2015).

It is well established that elderly individuals have increasing difficulties sleeping at night and have increase in daytime sleep episodes combined with generally going to sleep and waking up earlier (Kondratova and Kondratov, 2012). The daily cycles of hormone levels, body temperature, and the sleep-wake cycle are modified with age in humans, causing disruption in behavior and resultant reduction in the strength of the clock (Hofman and Swaab, 2006). Furthermore, circadian sleep-wake disorders are more prevalent in older individuals (Kim and Duffy, 2018).

Using *Drosophila* offers numerous advantages for investigating how aging affects the circadian clock, not least the strong history of circadian research in the model organism, genetic tractability, short lifespan (50–80 days), rapid generation time as well as clearly defined and manipulatable neural circuits. Genetic analysis of circadian behaviors has identified genes involved in generating rhythmic transcription translation feedback loops comprising the molecular clock of *Drosophila* (Allada and Chung, 2010, Hardin, 2011, Tataroglu and Emery, 2015), which in turn control a wide range of physiological and cellular responses, likely through rhythmic control of output genes.

The *Drosophila* central clock consists of 150 dispersed but connected circadian neurons that are classified by their anatomical location, projection pattern, and the expression of clock genes (Peschel and Helfrich-Förster, 2011). They function as a network to drive rhythmic behavior (Top and Young, 2018). Examples of outputs from the molecular clock are the circadian remodeling of the projections from the small ventral lateral clock neurons (s-LNvs) to the dorsal protocerebrum (Fernández et al., 2008) and circadian modulation of the firing frequency and membrane potential (MP) of clock neurons (Cao and Nitabach, 2008, Flourakis et al., 2015, Sheeba et al., 2008b). Under laboratory conditions using a 12:12 hours light:dark (LD) cycle, *Drosophila* display morning and evening peaks in locomotor activity with anticipation activity before the transitions of lights-on and lights-off, with constant darkness (DD) resulting in free-running activity with a period of around 23.8 hours (Dubowy and Sehgal, 2017).

The LNv neurons produce the neuropeptide pigment dispersing factor (PDF), which acts to synchronize activity throughout the clock circuit (Shafer and Yao, 2014). PDF acts through the PDF receptor that has broad expression in the circadian network (Im and Taghert, 2010), with rhythmic synaptic release of the PDF neuropeptide required for maintaining circadian rhythmicity under constant conditions. The PDF neurons have been termed the “morning” cells due to the absence of the morning (but not evening) peak of activity in flies either having mutations in the *pdf* gene or lacking the PDF neurons (Renn et al., 1999). Another group of clock neurons, the dorsal lateral neurons and the PDF-negative 5th s-LNvs, have been termed the “evening” cells as they are necessary for the evening anticipation activity (Grima et al., 2004, Stoleru et al., 2004).

In *Drosophila*, aging has been shown to cause reduced and weakened circadian activity in behavior, associated with declining levels of PDF (Umezaki et al., 2012). Disruption of the clock has also been shown to accelerate aging, in flies lacking a functional clock (Hendricks et al., 2003, Krishnan et al., 2012, Vaccaro et al., 2017), or keeping flies under mismatched lighting conditions (Klarsfeld and Rouyer, 1998, Pittendrigh and Minis, 1972, Vaccaro et al., 2016). Studies on how aging affects the molecular clock have reported conflicting results: it has been found to remain robust in aged flies (Luo et al., 2012) and to significantly decline in strength with age (Rakshit et al., 2012). To date, no studies have been published on the effect of aging on the electrical activity of clock neurons in *Drosophila*. In mice, aging has been shown to result in reduced amplitude of daily electrical rhythms, measured in vivo using multiunit recordings (Nakamura et al., 2011) or from single cells in slice preparations (Farajnia et al., 2012, Farajnia et al., 2015).

To investigate the effect of aging on circadian rhythms, we took advantage of the *Drosophila* model that allows systematic monitoring of circadian and sleep behavior simultaneous from flies across the range of lifespan. Furthermore, we determine the effect of aging at the neuronal activity level by making patch-clamp recordings from the large-LNv clock neurons from young and aged flies.

## 2 Materials and methods

### 2.1 Fly strains

The following fly stocks and their original sources were used: *Pdf*::*RFP* (Ruben et al., 2012), *iso31* (Ryder et al., 2004), *Pdf-Gal4* (Bloomington stock centre, #6900) (Park and Hall, 1998), and *UAS-mCD8*::*GFP* (Bloomington Stock Centre, #5137).

All flies were reared on a standard medium based on the following recipe: 10l batches containing 400 ml malt extract, 200 ml molasses, 400 g polenta, 90 g active dried yeast, 50 g soya flour, and 35 g granulated agar, with 40 ml of propionic acid (Sigma-Aldrich, #94425) and 100 ml of nipagin (Sigma-Aldrich, #H5501) added after cooling. Flies for aging were collected and flipped onto fresh food every 5 days and maintained in an incubator at 25 °C and humidity of 55%–65% with a 12:12 LD cycle.

### 2.2 Circadian behavior analysis

Locomotor activity of individual male flies (aged 1, 8, 15, 22, 29, 36, 43 and 49 days old) was measured using the *Drosophila* Activity Monitoring system (DAM2, TriKinetics Inc, USA). Flies were transferred into DAM tubes after reaching the desired age and were maintained for 5 days under 12:12 LD conditions, followed by DD. The first 7 days of DD activity was used for circadian analysis, with period and rhythmicity analysis performed in MATLAB using the Fly toolbox (Levine et al., 2002).

### 2.3 Anticipation index analysis

The morning and evening anticipation indexes were calculated from the activity of flies across the 5 days of LD activity. Morning anticipation was calculated as previously described (Harrisingh et al., 2007, Zhang and Emery, 2013). Briefly, the average activity was calculated as the ratio of activity between ZT21.5–24 and ZT17–19.5. Evening anticipation was likewise calculated as the ratio between ZT9.5–12 and ZT5–7.5.

### 2.4 Sleep analysis

Sleep data were analyzed using the Sleep and Circadian Analysis MATLAB Program (Donelson et al., 2012). Individual raster plots of activity were viewed, and flies that had died before the end of the experiment were removed from the data. Data were analyzed across the 24-hour period, the 12-hour “light phase” and the 12-hour “dark phase.” Sleep is visualized by plotting the mean amount of sleep in a 30-minute bin against the time of day, averaged for the 5 days of the experiment. From the raw data of sleep amounts and time, a series of measurements of sleep are calculated, including “total sleep duration”—sum of all sleep episodes, “number of sleep episodes”—count of all sleep episodes, “mean sleep episode duration”—average sleep duration (in minutes), and “sleep latency”—the time to the first sleep episodes (in minutes).

### 2.5 Electrophysiological recording of clock neurons

Whole-cell current clamp recordings were performed as previously described (Buhl et al., 2016, Chen et al., 2015). For visualization of the large ventral lateral neurons (l-LNvs), *Pdf*::*RFP* flies were used, which is a transgenic fusion of the *Pdf* promoter and *mRFP1* that specifically labels the LNv neurons. Adult male flies were maintained under a 12:12 LD cycle, and recordings were made at either ZT7-9 (day condition) or ZT19-21 (night condition), where ZT0 corresponds to lights-on.

First, flies were anesthetized using CO_2_, before decapitation, and the brain removed by acute dissection with fine forceps in extracellular saline solution containing (in mM) 101 NaCl, 1 CaCl_2_, 4 MgCl_2_, 3 KCl, 5 glucose, 1.25 NaH_2_PO_4_, and 20.7 NaHCO_3_ at pH 7.2. The photoreceptors, lamina, and as much membrane as possible were removed, and whole brains were transferred to a recording chamber (ALA Scientific) filled with extracellular solution and stably held ventral side up using a custom-built wire harp. Cells were visualized using an Axio Examiner Z1 (Zeiss) using a 63× water immersion objective, l-LNvs were identified using 555 nm light generated using a Colibri Examiner light source (Zeiss). All recordings were performed at room temperature (20 °C–22 °C) using thick-walled borosilicate glass electrodes (1B150F-4; World Precision Instruments) ranging in resistance from 10–16 MΩ filled with intracellular solution containing (in mM): 102 K-gluconate, 0.085 CaCl_2_, 17 NaCl, 0.94 EGTA, 8.5 HEPES, 4 Mg-ATP, and 0.5 Na-GTP at pH 7.2. Data were recorded using an Axon MultiClamp 700B amplifier, digitized with an Axon Digidata 1440A (sample rate 20 kHz, 10 kHz Bessel filter) and recorded using pClamp (10.5: Molecular Devices, CA, USA). Chemicals were acquired from Sigma (Poole, UK).

The liquid junction potential was calculated as 13 mV and subtracted post hoc from all the membrane voltages. A cell was included in the analysis if the access resistance was less than 50 MΩ. MP and the spontaneous firing rate (SFR) were measured after stabilizing for 2–3 minutes. Membrane input resistance (R_in_) was calculated by injecting hyperpolarizing current steps from −40 pA to +5 pA in 5 pA steps and measuring the resulting voltage change. Neuronal excitability was measured by injecting a 500 ms long positive current pulse in 2 pA increments with increasing amplitude up to +40 pA and manually counting the resulting spikes.

### 2.6 Immunohistochemistry and analysis

Flies were briefly anesthetized using CO_2_ and swiftly decapitated and heads immediately placed into phosphate-buffered saline (PBS) containing 4% paraformaldehyde (Image-iT Fixative Solution, Thermo Fisher Scientific # R37814) and 0.008% Triton X-100 (Sigma) and fixed for 45 minutes at room temperature. For all steps, tubes were covered by foil to protect tissue from light exposure. Fixed heads were quickly washed twice in 0.5% PBT (PBS with 0.5% Triton X-100) followed by three 20-minute washes in PBT, before being dissected in 0.1% PBT. Brains were blocked in 5% normal goat serum (NGS, Thermo Fisher Scientific # 50197Z) for 30 minutes at room temperature. Brains were then incubated with primary antibodies (Table 1) in 5% NGS at 4 °C for 36 hours on a rotator with tubes upright.

**Table 1:** Antibodies used and sources

Antibodies	Concentration	Source
Primary		
Mouse monoclonal anti-PDF	1:200	Developmental Studies Hybridoma Bank, #PDF-C7
Rabbit polyclonal anti-GFP	1:1000	Life Technologies # A11122
Secondary		
Alexa Fluor Plus 488 Goat anti-mouse	1:1000	Life Technologies # A32723
Alexa Fluor Plus 555 Goat anti-rabbit	1:100	Life Technologies # A32732

Brains were quickly washed twice in PBT, followed by three 20-minute washes in PBT, with tubes upright on a rotator. Brains were then incubated with secondary antibodies in 5% NGS for 3 hours at room temperature, and then overnight at 4 °C. Brains were rinsed in 0.1% PBT, followed by three 20-minute washes in PBT, and rinsed twice in PBS. Brains were then aligned on a microscope slide, with wells created using imaging spacers (SecureSeal, Grace Bio-Labs #654002), and then mounted in Vectashield hard set medium (Vector Laboratories). The mounting media was allowed to harden for 30 minutes at room temperature, before storage at 4 °C. Coverslip edges were sealed with clear solvent (CoverGrip, Biotium #23005).

Brains were imaged using a Leica TCS SP8 AOBS confocal laser scanning microscope attached to a Leica DMi8 inverted epifluorescence microscope, equipped with “hybrid” Gallium arsenide phosphide detectors with the green channel imaged at 480–551 nm and the red at 571–650 nm. We used a 20× glycerol immersion objective (HC PL APO CS2; Leica) and obtained confocal stacks with a 2 μm step size and 512 × 512 pixels. The obtained confocal stacks were analyzed using the Fiji implementation of ImageJ (Schindelin et al., 2012). Besides contrast, brightness, color scheme, and orientation adjustments, no further manipulations were made to the images.

To quantify the axonal arbor of the dorsal projections, we used an adaptation of the Sholl method (Sholl, 1953), as has been previously reported (Fernández et al., 2008). Briefly, using 6 evenly spaced (10 μm) concentric rings centered at the first branching of the dorsal projections and counting the number of intersections of each projection with the rings. Scoring was performed blind to the experimental condition.

### 2.7 Statistical analysis

All statistical analyses were performed using GraphPad Prism 7 (GraphPad Software, USA), with an α level of *p* < 0.05 considered significant. Data for aging experiments showed non-normal distribution and were plotted as the median with interquartile range; the nonparametric Kruskal-Wallis test was used, with post hoc analysis conducted using Dunn's test. For aging data, statistical comparisons between groups were compared to the D1 group.

For electrophysiological and imaging experiments, groups were compared using two-way ANOVA with Tukey's multiple comparisons test, with “age” and “time of day” as factors.

## 3 Results

### 3.1 Aging caused a weakening in circadian behavioral output and lengthening of the free-running period

**Fig. 1:**
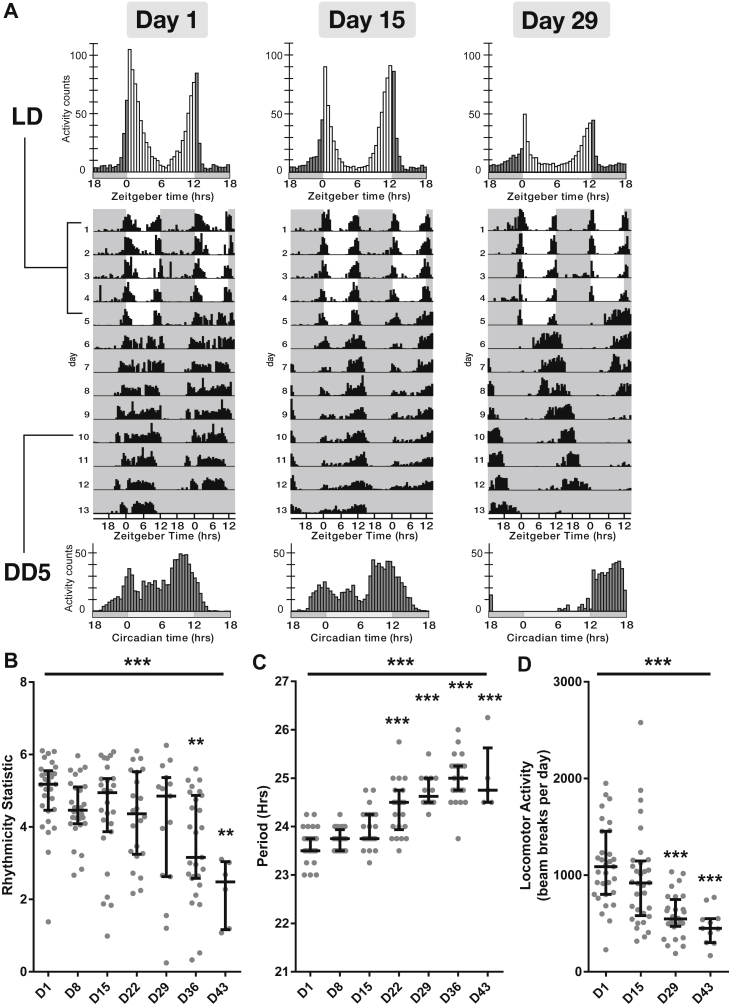
Locomotor activity at different time points in the aging process. (A) Top panel—group activity profiles during light:dark cycles (LD) from wild-type flies. Middle-panel—double-plotted actograms of representative individual flies are shown, activity scaled to individual maximum. Flies were maintained for 5 days of LD before being maintained in constant darkness (DD). In the actograms, white represents day and gray background represents darkness. Bottom panel—group activity profile during the 5th day of DD (DD5). (B) Circadian behavior weakens with age as measured by the rhythmicity statistic. (C) Aging causes lengthening of period in wild-type flies. (D) Average daily locomotor activity during LD is significantly reduced by aging. On the x axis, D signifies days after eclosion. ** representing *p* < 0.01, *** *p* < 0.001 as determined using the Kruskal-Wallis test with Dunn's post hoc test; data plotted as median with error bars representing the interquartile range.

To address the impact of aging on the circadian clock, we used *Drosophila* to conduct a comprehensive behavioral analysis of circadian activity of male flies at 1-week intervals during the aging process. Flies were first kept for 5 days in a 12 hours:12 hours LD cycle and showed normal diurnal behavior, with morning and evening peaks of activity (Fig. 1A). Flies were then maintained in DD and showed typical free-running behavior. In agreement with previously reported work (Rakshit et al., 2012, Umezaki et al., 2012), we found that aging resulted in a significant decline in the strength of circadian locomotor activity under free-running conditions (Fig. 1B, *p* = 0.0001, Kruskal-Wallis statistic = 27.17), with a steep decline in flies aged 36 days and older and a significant lengthening of the period of the observed behavioral activity (Fig. 1C, *p* < 0.0001, Kruskal-Wallis statistic = 95.3). There we also found a significant age-related reduction in total locomotor activity (Fig. 1D, *p* < 0.0001, Kruskal-Wallis statistic = 37.89).

### 3.2 Aging alters daily activity structure in light-dark conditions and reduces anticipatory behavior

**Fig. 2:**
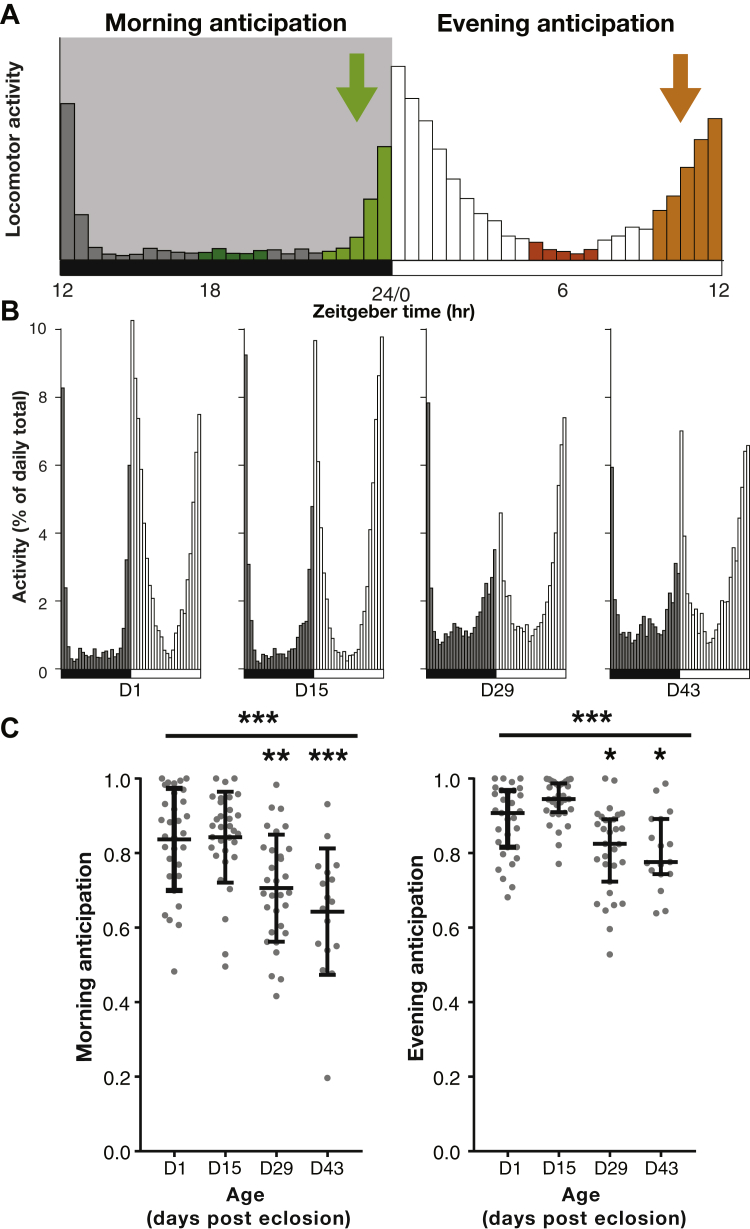
Morning and evening anticipation reduce with age in wild-type flies. (A) Schematic of morning and evening anticipation index. (B) Normalized daily group activity plots of 1-, 15-, 29-, and 43-day-old flies. (C) Quantification of anticipation index shows that the morning anticipation index is significantly reduced by age and that evening anticipation is slightly reduced. N = 20–32 for each group, * represents *p* < 0.05, ** *p* < 0.01, *** *p* < 0.001 as determined using the Kruskal-Wallis test with Dunn's post hoc test; data plotted as median with error bars representing the interquartile range.

Given that there was a reduction in the amount of locomotor activity in older flies (Fig. 1D), we sought to further examine how the daily structure of activity under normal LD conditions was altered by the aging process. Some of the hallmark features of daily activity of male flies recorded using the DAM system are the morning and evening peaks in locomotor activity (Dubowy and Sehgal, 2017) and anticipation of the light-dark transition (see Fig. 2A). Both the peaks of activity and anticipation behavior are affected by manipulations of the circadian system (Lear et al., 2009). We investigated the effect of age on morning and evening anticipation by first normalizing locomotor activity for an individual fly to be the percentage of the daily total (Fig. 2B). The anticipation index was then quantified as the proportion of an individual fly's daily activity occurring in the 2.5 hours immediately before the LD transition compared with the 2.5 hours in the middle of the day/night (Harrisingh et al., 2007). Older flies showed a significant reduction in morning anticipation index compared with young flies (*p* < 0.0001, Kruskal-Wallis statistic = 28.93), and a slight reduction in evening anticipation index (*p* < 0.0001, Kruskal-Wallis statistic = 32.45) (Fig. 2C).

### 3.3 Aging alters the daily structure of sleep

Given that there is a strong connection between the circadian clock and sleep, we sought to also investigate how sleep is altered by age. Sleep analysis was performed for the 5 days under an LD cycle at the start of the circadian experiment, with sleep classified under the common convention of periods of immobility longer than 5 minutes in duration (Hendricks et al., 2000, Shaw et al., 2000).

**Fig. 3:**
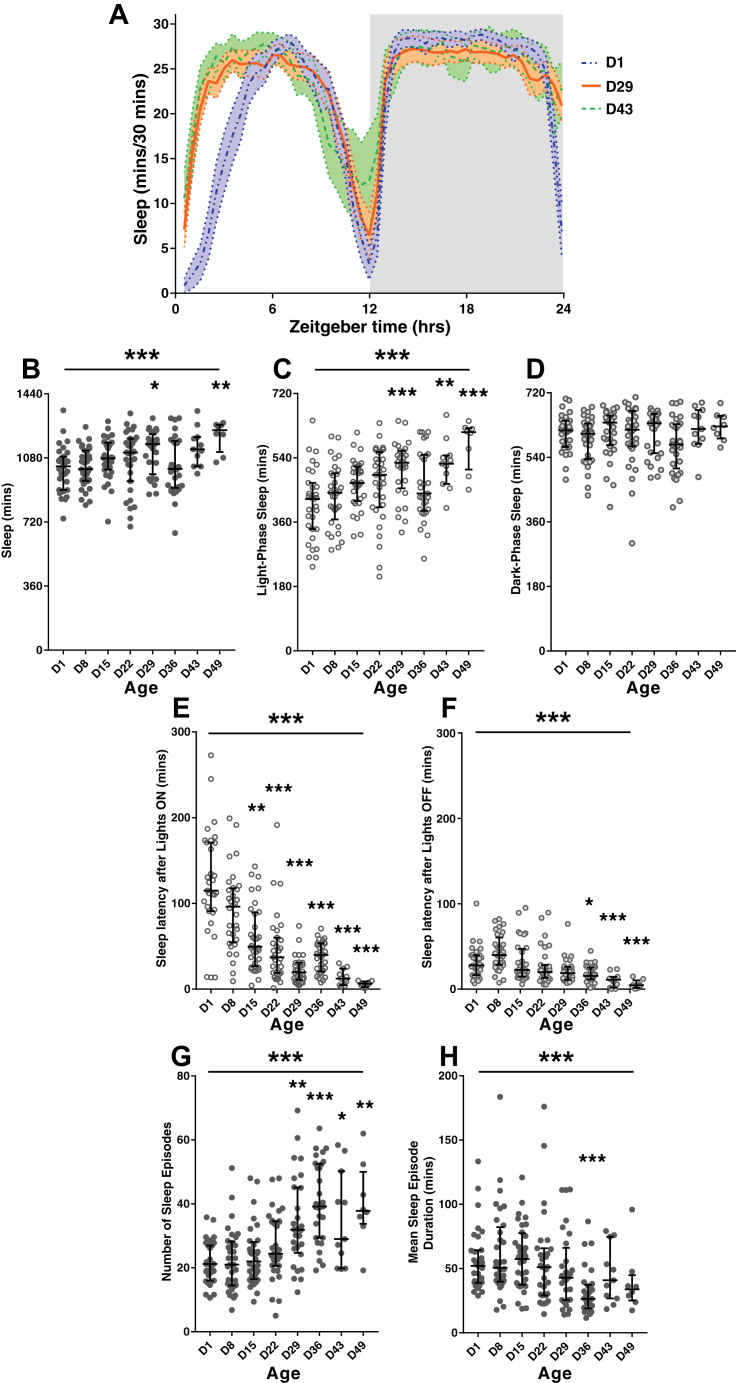
Aging alters the daily structure of sleep in *Drosophila*. (A) Daily sleep profile of groups of male flies aged 1, 29, and 43 days, average across 5 days. Shaded area represents the 95% confidence interval. (B–H) Quantification of sleep parameters for flies aged D1, 8, 15, 22, 29, 36, 43, and 49 days; flies were monitored in parallel under identical conditions. Error bars plot the median and interquartile range with * representing *p* < 0.05, ** *p* < 0.01, *** *p* < 0.001 with statistical testing performed by Kruskal-Wallis test with Dunn's multiple comparisons. (B) Mean total sleep duration; (C) mean daytime sleep; (D) mean night-time sleep; (E) latency to sleep after lights-on; (F) latency to sleep after lights-off; (G) number of sleep episodes; (H) sleep episode duration (see Methods for definitions).

The daily structure of sleep in older male flies was noticeably different to that of young flies (Fig. 3A) with a visible increase in the amount of daytime sleep and a shift toward sleep earlier in the day. Quantification of total sleep showed a significant effect of age (*p* = 0.0006, Kruskal-Wallis statistic = 25.7) (Fig. 3B). Looking only at sleep during the daytime (Fig. 3C), there was a significant increase with age (*p* < 0.0001, Kruskal-Wallis statistic = 33.83); however, there was no effect of age on night-time sleep (*p* = 0.31 Kruskal-Wallis statistic = 8.253) (Fig. 3D). Measuring the latency of sleep after the LD transitions demonstrated a significant reduction in the speed at which older flies started sleeping both during the day (*p* < 0.0001, Kruskal-Wallis statistic = 102.7) (Fig. 3E) and night (*p* < 0.0001, Kruskal-Wallis statistic = 61.33) (Fig. 3F). Analyzing the parameters of sleep episodes, we found that there was a significant increase in the number of sleep episodes with age (*p* < 0.0001, Kruskal-Wallis statistic = 58.79) (Fig. 3G) and a significant difference in mean sleep episode duration (*p* < 0.0001, Kruskal-Wallis statistic = 32.56) (Fig. 3H).

### 3.4 Electrical properties of clock neurons are altered by aging

**Fig. 4:**
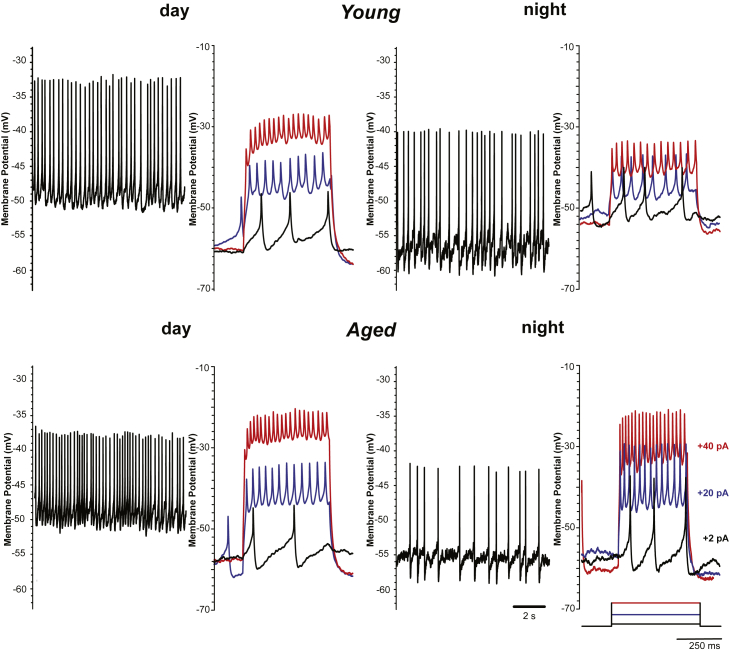
Electrophysiological characterization of l-LNv clock neurons. Membrane potential and spontaneous activity (left panels) and firing response to a current pulse (right panels, color-coded as indicated) of wild-type l-LNvs from young (day 1–5) and aged (day 28–35) flies recorded at day (ZT 7–9) and night (ZT 19–21). Abbreviation: l-LNv, large ventral lateral neurons. (For interpretation of the references to color in this figure legend, the reader is referred to the Web version of this article.)

**Fig. 5:**
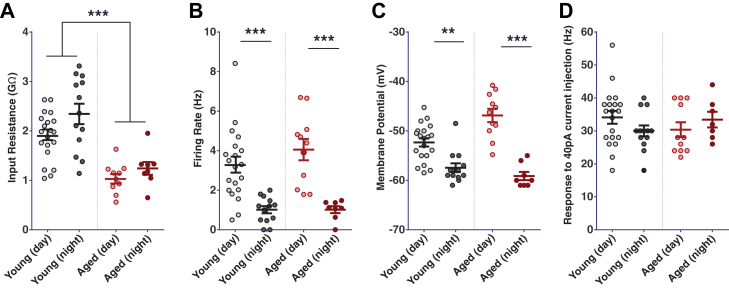
Quantitative analysis of electrophysiological properties of large lateral ventral clock neurons (l-LNv) from young (day 1–5) and middle-aged (day 28–35) flies in day and night conditions. (A) Analysis of input resistance (R_in_) showed a highly significant effect of age (*p* < 0.0001) and an effect of time of day (*p* < 0.005) but no interaction. (B) Analysis of the spontaneous firing rate (SFR) showed a significant effect of time of day (*p* < 0.0001) but no effect of age. (C) Analysis of the membrane potential (MP) values again showed only a significant effect of time of day (*p* < 0.0001) but not age. (D) Analysis of the responses to an injected current pulse (f_+40pA_) showed no significant effects. Data were analyzed using two-way ANOVA with Tukey's multiple comparisons test, ** representing *p* < 0.01, *** *p* < 0.001, error bars show the mean ± SEM. Each data point represents a single l-LNv neuron.

We have demonstrated that the aging process causes significant changes to the behavioral outputs of the circadian clock circuit of *Drosophila* and therefore set out to investigate if these were underpinned by changes in clock neuronal activity. To measure the effects of aging on clock neurons, we made recordings from the prominent wake-promoting and arousal l-LNv neurons, the most accessible and well-studied group of clock neurons in *Drosophila* (Buhl et al., 2016, Cao and Nitabach, 2008, Parisky et al., 2008, Sheeba et al., 2008a). Recordings were made during the day and at night in explant brain preparations made from young (day [d] 1–5) and middle-aged (d28-35) flies (Fig. 4) and measured the electrophysiological properties of these cells (Fig. 5). Recordings from flies older than 35 days were limited due to the technical difficulties making stable recordings from aged neurons, with older brains being more difficult to dissect cleanly and difficulties to achieve good seals and access due to changes in the older membranes.

As previously reported, young l-LNvs showed a strong day-night difference in both their SFR and MP (Buhl et al., 2016, Chen et al., 2015, Sheeba et al., 2008b), but the response to an injected current pulse or the input resistance did not differ significantly between day and night (Fig. 5). Here we report that the diurnal modulation of SFR and MP are maintained in the l-LNvs recorded from 28- to 35-day-old flies, with no difference found in the response to a current injection between young and aged flies. Interestingly, we report a significant decrease in the input resistance of aged l-LNvs (Fig. 5D), indicative of an increase in overall conductance across the membrane.

### 3.5 s-LNv terminal remodeling is reduced by aging

**Fig. 6:**
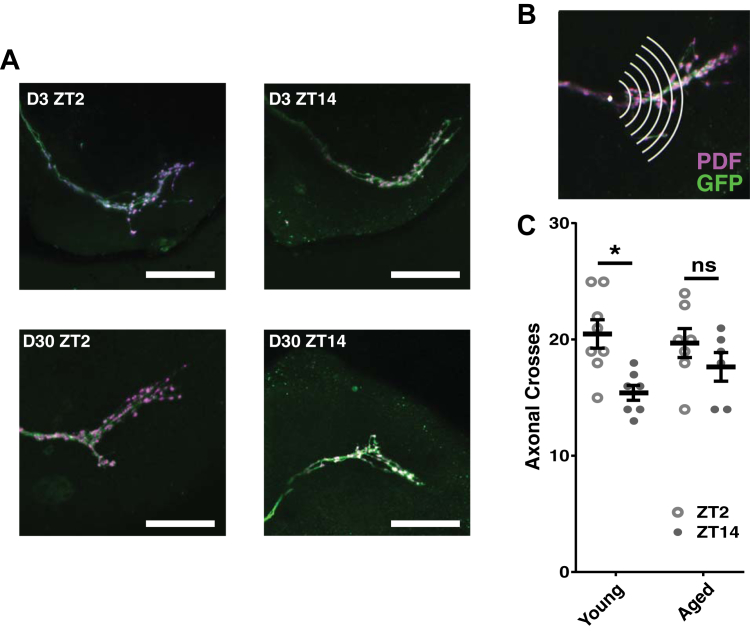
Daily reorganization in the PDF terminals is reduced by aging. (A) *pdf* > *mCD8-GFP* wild-type brains dissected at ZT2 and ZT14. Brains were stained with anti-GFP (green) and anti-PDF (magenta) antibodies. Scale bar = 50 μm. (B) Schematic depiction of how the quantification of the PDF axonal branching was carried out. (C) The total number of intersections between the concentric rings and the axonal projections was quantified and showed daily remodeling. Error bars show mean ± SEM, statistical analysis performed by two-way ANOVA with Tukey's multiple comparisons test, * represents *p* < 0.05. N > 6 for all groups; quantification was performed on the dorsal projections originating from a group of s-LNv neurons. Abbreviations: PDF, pigment dispersing factor; s-LNv, small ventral lateral neuron. (For interpretation of the references to color in this figure legend, the reader is referred to the Web version of this article.)

It has previously been demonstrated that the dorsal projections of the s-LNv neurons show daily remodeling in complexity under clock control (Fernández et al., 2008). To test if this was still occurring in older flies, day/night changes in PDF terminal morphology were measured in flies aged 30 days (Fig. 6). Using the previously published protocol (Fernández et al., 2008), we found that the remodeling was no longer a significant feature in aged brains. There was a significant overall effect of time of day (*p* = 0.0045, two-way ANOVA, F(1,24) = 9.822), but no effect of age (*p* = 0.5286, two-way ANOVA, F(1,24) = 0.4088). Multiple comparisons tests showed the magnitude of the day-night difference was reduced in older flies and no longer being significantly different between day and night (*p* = 0.6138, Tukey's test, DF = 24, q = 1.741) (Fig. 6C).

## 4 Discussion

Aging is known to have a significant impact on circadian behavior but what effect this has at a neuronal level is poorly understood. In this study, we have conducted a systematic analysis of the effect of aging on circadian behavior and related this to electrical activity of l-LNv clock neurons finding a significant reduction in the input resistance of aged neurons.

Our behavioral experiments complement the work of previous studies in showing that the strength of the free-running behavior weakens and period lengthens with age (Umezaki et al., 2012). In addition, we go further by using a systematic approach to monitor flies at 1-week intervals across the aging process and show that there is an age-dependent decrease in rhythm strength (Fig. 1B) and an equivalent increase in period length with age (Fig. 1C). Mouse experiments have found that aging results in a lengthening in period in both behavioral activity (Turek et al., 1995, Valentinuzzi et al., 1997) and molecular rhythms in the suprachiasmatic nucleus (Nakamura et al., 2015).

We further sought to investigate how the daily structure of activity under LD conditions is altered by aging, by quantifying changes in morning and evening anticipatory activity (Fig. 2). We found that there was a significant effect of age on both the morning and evening anticipation indexes (Fig. 2C), with a greater reduction in the morning peak. LNv neurons are required for correct morning anticipation (Grima et al., 2004, Stoleru et al., 2004) and are obvious candidates for involvement in an age-related decline in this anticipatory behavior. Morning anticipatory behavior is also linked to expression of PDF, with *pdf* ^*01*^ and *PDF-RNAi* flies showing significant reductions in morning anticipation (Shafer and Taghert, 2009). A reduction in PDF expression in aged flies has previously been demonstrated (Umezaki et al., 2012), providing further evidence for the importance of PDF in maintaining healthy rhythms with age and supporting a hypothesis that reduced PDF signaling with age underlies the weakening of behavioral rhythmicity.

The l-LNv neurons are involved in promoting arousal (Chung et al., 2009, Sheeba et al., 2008a) and regulating sleep and latency during the early night (Liu et al., 2014). We made use of the DAM recording system to monitor sleep under LD conditions, using the widely accepted definition of sleep as period of immobility greater than 5 minutes (Shaw et al., 2000). Aging is known to cause changes in the sleep profile across many organisms including mice (Valentinuzzi et al., 1997), nonhuman primates (Zhdanova et al., 2011), and humans (Moraes et al., 2014). Previous *Drosophila* studies on the effects of aging on sleep have reported that sleep becomes more fragmented with age (Koh et al., 2006, Vienne et al., 2016), showing a similar increase in sleep episode number and decrease in mean sleep episode duration compared with our results (Fig. 3G and H).

Electrical silencing of LNv neurons causes deficits in free-running clock behavior (Depetris-Chauvin et al., 2011), demonstrating a link between electrical activity and behavior. Most electrophysiological studies use young flies aged between 3 and 7 days for recordings (Cao and Nitabach, 2008), with a limited amount of recordings made from 25-day-old flies only looking at the active firing properties of the neurons (Sheeba et al., 2008b). Here we report the effect of aging on l-LNv neuronal activity and electrical properties. We perform whole-cell patch clamp recordings from young and aged neurons and report no major differences in the observed spontaneous activity of l-LNv neurons (Fig. 4). Further analysis of the electrical properties of l-LNv neurons showed that there was a significant effect of age in reducing the input resistance, which surprisingly did not affect SFR, MP or excitability (Fig. 5). We propose the age-related changes in l-LNv properties are linked with the observed changes in activity and sleep during light-dark conditions.

There are multiple possible explanations for a decrease in input resistance without changing the active properties of the neurons. One hypothesis would be the involvement of chloride (Cl^−^) channels, which could become open and decrease resistance without changing the MP, alternatively, the observed reduction in input resistance could result from age-related changes in the composition of ion channels in the membrane, with future experiments needed to evaluate between potential hypotheses. The l-LNv express the GABA_A_ receptor *Resistant to dieldrin* (*Rdl*), which when activated by GABA selectively conducts Cl^−^ through its pore. *Rdl* has important roles in promoting sleep, with a mutation in *Rdl* that causes extended channel openings resulting in increased sleep duration and decreased latency (Agosto et al., 2008, Parisky et al., 2008). Conversely, knocking down the *Rdl* gene in the PDF neurons reduces sleep, again suggesting GABA regulates sleep through the LNvs and *Rdl* receptor function (Chung et al., 2009). Therefore, it is possible that during aging, there is an increase in GABA activation through *Rdl* in the l-LNvs, causing increased Cl^−^ conductance. This increase in Cl^−^ conductance may contribute to the observed reduction in input resistance recorded and also drive the increase in sleep duration and decreased sleep latency in aged flies.

Studies of aging on electrical activity of mouse clock neurons found no effect of age on input resistance but reveal a reduction in the difference between day and night firing rates (Farajnia et al., 2012), showing differences of the effects of aging between different clock neurons in *Drosophila* and mouse.

Our electrophysiological experiments were limited to the l-LNvs, so we can only link the changes in neuronal properties we observed to the changes in morning activity and sleep in LD conditions as the l-LNv do not maintain molecular oscillations in DD (Grima et al., 2004), although it is possible that similar changes in neuronal properties are occurring in the s-LNvs where molecular oscillations do persist in constant conditions. We sought to investigate changes to the s-LNv neurons, namely the remodeling of the s-LNv dorsal projections. Analysis of the branching of the s-LNv projections demonstrated that the day-night difference in complexity is reduced by aging (Fig. 6), indicating changes in the distribution of the PDF release network in older flies. Given the role of PDF in regulating the activity of different groups of clock neurons, namely through excitation of dorsal clock neurons (Seluzicki et al., 2014), changes in PDF signaling would contribute to changes in the clock network as a whole. The s-LNv neurons are known to be important for maintaining behavioral rhythmicity under constant conditions, and we propose this weakening of s-LNv terminal remodeling underlies the age-related weakening in circadian locomotor behavior.

Our study builds upon the existing literature demonstrating an age-dependent decline in circadian behavioral outputs and importantly links this to changes in the electrophysiological and structural properties of clock neurons. Further work is necessary to fully understand what the implications of these changes are for the circadian clock network as a whole and if similar changes are occurring in other groups of clock neurons in *Drosophila*.

## Disclosure

The authors have no actual or potential conflicts of interest.
